# Unique Pharmacological Properties of α-Conotoxin OmIA at α7 nAChRs

**DOI:** 10.3389/fphar.2021.803397

**Published:** 2021-12-08

**Authors:** Thao N.T. Ho, Nikita Abraham, Richard J. Lewis

**Affiliations:** Centre for Pain Research, Institute for Molecular Bioscience, The University of Queensland, St Lucia, QLD, Australia

**Keywords:** conotoxins, ligand-gated ion channels, α7 nAChR, pharmacology, AChBP, X-ray structrures

## Abstract

OmIA, isolated from *Conus omaria* venom, is a potent antagonist at α7 nAChRs. We determined the co-crystal structure of OmIA with *Lymnae stagnalis* acetylcholine binding protein (*Ls*-AChBP) that identified His5, Val10 and Asn11 as key determinants for the high potency of OmIA at α7 nAChRs. Remarkably, despite a competitive binding mode observed in the co-crystal structure, OmIA and analogues displayed functional insurmountable antagonism at α7 and α3β4 nAChRs, except OmIA analogues having long side chain at position 10 ([V10Q]OmIA and [V10L]OmIA), which were partial insurmountable antagonist at α7 nAChRs in the presence of type II positive allosteric modulators (PAMs). A “two-state, two-step” model was used to explain these observations, with [V10Q]OmIA and [V10L]OmIA co-existing in a fast reversible/surmountable as well as a tight binding/insurmountable state. OmIA and analogues also showed biphasic-inhibition at α7 nAChRs in the presence of PNU120596, with a preference for the high-affinity binding site following prolonged exposure. The molecular basis of binding and complex pharmacological profile of OmIA at α7 nAChRs presented in here expands on the potential of α-conotoxins to probe the pharmacological properties of nAChRs and may help guide the development novel α7 modulators.

## Introduction

α-Conotoxins are among the smallest conopeptides from *Conus* venoms (12–20 amino acids (aa)). Classical α-conotoxins are characterised by a CC–X_m_–C–X_n_–C framework forming three possible disulfide connectivities: globular (I–III, II–IV), ribbon (I–IV, II–III) and bead (I–II, III–IV) (Azam and Mcintosh, 2009; Lewis et al., 2012; Ho et al., 2020) with the globular conformation generally the native bioactive isomer. α-conotoxins are further divided into several structural subgroups (*m/n*: 3/5, 5/5, 4/3, 4/4, 4/5, 4/6 and 4/7) based on the number of residues within the two loops (*m*, *n*) braced by the disulfide bonds. The pharmacological targets tend to correlate to the loop size, with α3/5-conotoxins active toward muscle nAChRs, while the 5/5, 4/3, 4/4, 4/5, 4/6 and the most common 4/7 subgroup mainly interacting with neuronal nAChRs (Abraham and Lewis, 2018; Jin et al., 2019). The exquisite potency and selectivity of these peptides have helped build our understanding of the pharmacology of nAChRs.

nAChRs are prototypical ligand-gated ion channels found in the central and peripheral nervous system, and recently in other biological systems such as in immune system, muscles, skin and lung (Taly and Charon, 2012). This family constitutes interesting therapeutic drug targets due to its association with the progression of CNS disorders (Gotti and Clementi, 2004; Hogg and Bertrand, 2004; Dani and Bertrand, 2007). Neuronal nAChRs are assembled as homopentamers of α7, α8 and α9 or heteropentamers of α2–α6 in complex with β2–β4 or α7 with β2 subunits or α9 with α10 subunits. Among different nAChRs subtypes, the homopentameric α7 nAChR is one of the most abundant nAChRs in the nervous system and also in many non-neuronal cells (Taly and Charon, 2012). It exhibits unique characteristics, including high permeability to Ca^2+^, low agonist sensitivity in the resting state, inotropic/metabotropic dual action, rapid activation and fast desensitization. Agonist activation of α7 nAChRs is also sensitive to modulation by positive allosteric modulators (PAMs), with type I PAMs potentiating peak agonist responses and type II PAMs prolonging the opening times of nAChR by reducing receptor desensitization (Bertrand and Gopalakrishnan, 2007; Taly et al., 2009). These properties together with its involvement in pathologic conditions and the therapeutic potential of α7 ligands have made α7 nAChRs an important emerging drug targets (Lendvai et al., 2013; Dineley et al., 2015; Corradi and Bouzat, 2016).

OmIA, α-conotoxin purified from *Conus omaria,* is an α4/7-conotoxins that competitively antagonises α7 nAChRs (Talley et al., 2006). In this paper, we present the co-crystal structure of OmIA with *Lymnae stagnalis* (*Ls*)-acetylcholine binding protein (AChBP) that revealed three residues, His5, Val10 and Asn11, play essential roles in the activity of OmIA at α7 nAChRs. Interestingly, despite a competitive binding mode observed in the OmIA/*Ls*-AChBP complex, OmIA and analogues for the first time were found to display functional insurmountable antagonism at α7 and α3β4 nAChRs, with OmIA analogues having long side chains at position 10 acting as partial insurmountable antagonists at α7 nAChRs in the presence of type II PAMs. These studies provide with new insights into the pharmacological properties of α-conotoxins as well as the important α7 nAChR subtype.

## Materials and Methods

### Peptide Synthesis by Two-step Oxidation

α-Conotoxin OmIA and its variants were assembled by solid-phase methodology on a Liberty PRIME peptide synthesizer (CEM, United States) using Fmoc chemistry and standard side chain protection, except for cysteine residues. Cys residues were orthogonally protected in pairs with acid-labile trityl (Trt) and acid-stable S-acetamidomethyl (Acm) respectively on Cys^I^-Cys^III^ and Cys^II^- Cys^IV^ for globular isomer. Peptide cleavage from resin and global side chain deprotection were done by treatment with scavenger mixture (trifluoroacetic acid (TFA)/water/triisopropylsilane, 95:2.5:2.5, v/v/v) for 30 min at 40°C on Razor system (CEM, United States). The cleaved peptides were precipitated with cold diethyl ether, centrifuged (1957x*g* x3), redissolved in 50% aqueous acetonitrile (ACN) and lyophilized.

Disulfide bonds were formed selectively via a directed two-step oxidation. Trt protecting groups of the first Cys pairs were removed after peptide cleavage from resin, while Acm groups on the second Cys pairs remained intact. The oxidation of peptides was carried out in a buffer of 90% acetic acid/10% methanol (MeOH) with peptides at final concentration of 2 mg/ml. The first disulfide bridge formation between free cysteines was performed upon the dropwise addition of iodine (I_2_) (10 mg/ml dissolved in MeOH) while stirring until a pale yellow colour persisted. The solution containing partially oxidized peptide was then diluted with an equal volume of 50 mM HCl in 50% aqueous MeOH. Simultaneous removal of the Acm group and the second disulfide bridge formation were accomplished by the continued addition of I_2_ (8 equivalents). The oxidation reaction was monitored via analytical high performance liquid chromatography (a linear gradient of 10–40% solvent B (90%ACN/0.05%TFA) over 30 min at a flow rate of 1 ml/min on a C18 column (Vydac 218 TP, Grace) and electrospray ionization-mass spectrometry. The oxidation reaction was quenched by the addition of ascorbic acid and diluted 20-fold with solvent A. Bicyclic peptide was purified by reverse phase- high performance liquid chromatography (RP-HPLC) on a C18 Vydac column (Vydac 218 TP, Grace) using a linear gradient of 5–45% solvent B over 40 min at a flow rate of 16 ml/min. The final product was collected and analyzed by analytical HPLC and matrix-assisted laser desorption/ionization-time of flight.

### AChBPs Protein Expression and Purification

The over-expression of *Ls*-AChBP was performed as previously described (Abraham et al., 2016). Ubiquitin (Ub)-tagged AChBPs were used for radioligand binding assay. De-tagged *Ls*-AChBP was used for crystallization. Briefly, after immobilized metal affinity chromatography purification (IMAC), *Ls*-AChBPs were removed from Ub-tag by DUB enzyme (produced in-house). De-tagged *Ls*-AChBP was further purified by size exclusion chromatography to assess homogeneity and oligomerization state. The IMAC purified *Ls*-AChBP was analyzed on a calibrated analytical HiLoad 16/600 column and (GE Health care) for *Ls*-AChBP respectively using AKTA fast protein liquid chromatography system (GE Health care). The fractions containing the proteins were pooled and concentrated to the desired concentration using an Amicon centrifuge filter (30-kDa cut-off, Millipore).

### Circular Dichroism Analysis

Circular dichroism (CD) analysis was used to study the secondary structure of peptides. CD spectra were obtained from Jasco J-810 spectropolarimeter (Jasco, Tokyo, Japan). Peptides were dissolved in 20 mM ammonium bicarbonate buffer pH 7.4 and 55% trifluoroethanol at a final concentration of 50 μM. All measurements were done at room temperature under a nitrogen atmosphere (15 ml/min) with a scanning speed at 10 min and a 4 s response time. Absorbance was measured in the far-UV region (185–260 nm) via a cell with a path length of 1 cm and the capacity of 400 μL. Interference due to solvent, cell, or spectropolarimeter optics was eliminated via the subtraction of CD spectra of the pure solvents from those of the peptide. CD data in ellipticity was calculated to mean residue ellipticity [(θ)] using the equation: (θ) = θ/[10 × Np × (1,000 × Np × C) × l], where θ is the ellipticity in millidegrees, C is the peptide molar concentration (M) of the peptide, l is the cell path length (cm), and Np is the number of peptide residues.

### Crystallization and Data Collection

Purified de-tagged *Ls-*AChBP and OmIA were mixed at a molar ratio of 1:2 at 4°C for 1 h before setting up crystallisation trials. Crystals were successfully grown at room temperature using the hanging drop method by mixing volumes of protein and reservoir solution at a ratio 2:1. The crystals for OmIA were grown at 0.2 M ammonium sulfate, 5% PEG4000 and 0.1 M sodium acetate trihydrate at pH 4.6. The crystals were cryo-protected with reservoir solution plus 20% (v/v) glycerol in liquid nitrogen.

### Structure Determination and Refinement

Diffraction data were collected at the MX2 beam line of Australian Synchrotron, Melbourne. Diffraction data were indexed, integrated via XDS and Molfsm and scaled via AIMLESS (Collaborative Computational Project, Number 4, 1994; Battye et al., 2011). The structure was solved via molecular replacement using the PHASER (Mccoy et al., 2007) crystallographic software with LsIA/*Ls*-AChBP (PDB 2C9T) as search model. Refinement against experimental data was done using Phenix. refine and COOT until clear electron densities for OmIA were visible (Emsley and Cowtan, 2004; Afonine et al., 2012). NCS restraints and TLS restrains were then applied and the final structures validated with MOLPROBITY and PDB validation (Chen et al., 2010).

### Homology Modelling

The homology modellings were performed via the project mode of the SWISSMODEL online server (Guex et al., 2009). Briefly, the homology models were generated via the alignment of the ligand binding domain of the nAChRs with the crystal structure of the OmIA with *Ls*-AChBP. The quality of alignment was manually adjusted. The resulting model was energy minimized using the GROSMACS force filed in the program DEEPVIEW and models were analyzed in PyMol.

### Cell Culture

Cell culture was performed as previously described (Inserra et al., 2013). Briefly, SH-SY5Y neuroblastoma cells (a gift from Victor Diaz, Max Plank Institute for Experimental Medicine, Goettingen, Germany) were cultured at 37°C/5% (v/v) CO_2_ in RPMI media containing 2 mM L-glutamine and 15% (v/v) FBS. Cells were passaged every 3–5 days using 0.25% trypsin/EDTA at a dilution of 1:5. Experiments were conducted over several months and spanned on average a minimum of 10–20 passages. Responses were not affected by cell passage number with consistent control responses recorded over the duration of experiments as responses.

### FLIPR Assay

FLIPR assay was performed as previously described (Inserra et al., 2013). Briefly, cultured SH-SY5Y cells were plated at a density of 100,000 cells per well on black-walled 384-well imaging plates and cultured for 48 h to form a confluent monolayer. Growth media was removed and incubated for 30 min at 37°C with component A of the Calcium 4 assay kit. Intracellular increases in calcium in response to choline activating α7 nAChRs endogenously expressed by the SH-SY5Y cells. After incubation, the cells were transferred to the FLIPR (Molecular Devices). The changes in fluorescence correlated to intracellular calcium levels were measured using a cooled CCD camera with excitation 470–495 nm, emission 515–575 nm every 1s. Camera gain and intensity were adjusted for each plate of cells yielding a minimum of 1,500–2000 arbitrary fluorescence units (AFU) as a baseline fluorescence value. OmIA and analogues were added 10 min before applying choline for α7 nAChRs (30 μM). N-(5-Chloro-2,4-dimethoxyphenyl)-N′-(5-methyl-3-isoxazolyl)-urea (PNU120596) and TQS were also used (10 μM) to measure activity at the α7 subtype on the FLIPR platform. The channel kinetics are too fast to measure otherwise. All compounds were diluted with physiological salt solution [PSS (mM); 5.9 KCl, 1.5 MgCl_2_, 1.2 NaH_2_PO_4_, 5.0 NaHCO_3_, 140 NaCl, 11.5 glucose, 5 CaCl_2_, 10 HEPES at pH 7.4]. FLIPR data was normalised to the maximum choline response in the SH-SY5Y cells to yield the %Fmax. A four-parameter Hill equation was fitted to the data using GraphPad Prism 9.0. For the examination of biphasic behaviours, a biphasic model was fitted to the data using GraphPad Prism 9.0. Experiments were performed in triplicates in three independent experiments. IC_50_ and EC_50_ values are reported as means ± S.E.M.

### Binding Assays

The ability of VxXIIB variants to displace the binding of [^3^H]-epibatidine to the recombinantly expressed *Ls*-AChBP was determined in competitive radioligand binding assays. Briefly, [^3^H]-epibatidine (1 nM final concentration) and increasing concentrations of test ligand in a final volume of 100 ml were incubated in 96-well plates (Flexible PET Microplate, Perkin Elmer) precoated with 1 ng/ml of *Ls*-AChBP per well in binding buffer (phosphate buffered saline with 0.05% bovine serum albumin). The mixture was then removed and 100 ml of scintillant (Optiphase Supermix, Perkin Elmer) added to each well. Bound radioactivity was measured with a Wallac 1450 MicroBeta liquid scintillation counter (Perkin Elmer).

### Data Analysis

Comparison of the IC_50_ values of each analogue with the wildtype α-conotoxin and comparison of agonist EC_50_ values in the presence of fixed concentration of antagonist with agonist EC_50_ values in the absence of antagonist were carried out by pairwise comparison using an extra sum-of-squares F test with *p* < 0.05 in GraphPad Prism 9.0. Statistical analysis for partial inhibition of the concentration-response curves, where a fixed concentration of agonist was added to an increasing concentration of antagonist, was determined as significant if 95% confidence interval (CI) of curve bottom values did not overlap 0%. Statistical analysis for insurmountable inhibition, where a fixed concentration of antagonist was added to an increasing concentration of agonist, was determined as significant if the 95% CI for the curve top values of the concentration-response did not overlap 100%.

## Results

### Crystal Structure of OmIA in Complex With *Ls*-AChBP

The structure of OmIA and *Ls*-AChBP was solved at 2.47 Å resolution (Supplementary Table S1). The diffraction data and electron density map were well defined, except certain residues on the flexible loops (mostly AChBP loop F: Thr156, Asn158, Ser159, Asp160) were excluded from the final model due to their ambiguous electron density. The crystals belonged to space-group P42_1_2 and had the following unit cell dimensions: a = 169.4 Å, b = 169.4 Å, c = 124.3 Å. The structure was determined by molecular replacement with the structure LsIA/*Ls*-AChBP as the model and refined to a R_free_ value of 0.247.

**FIGURE 1:**
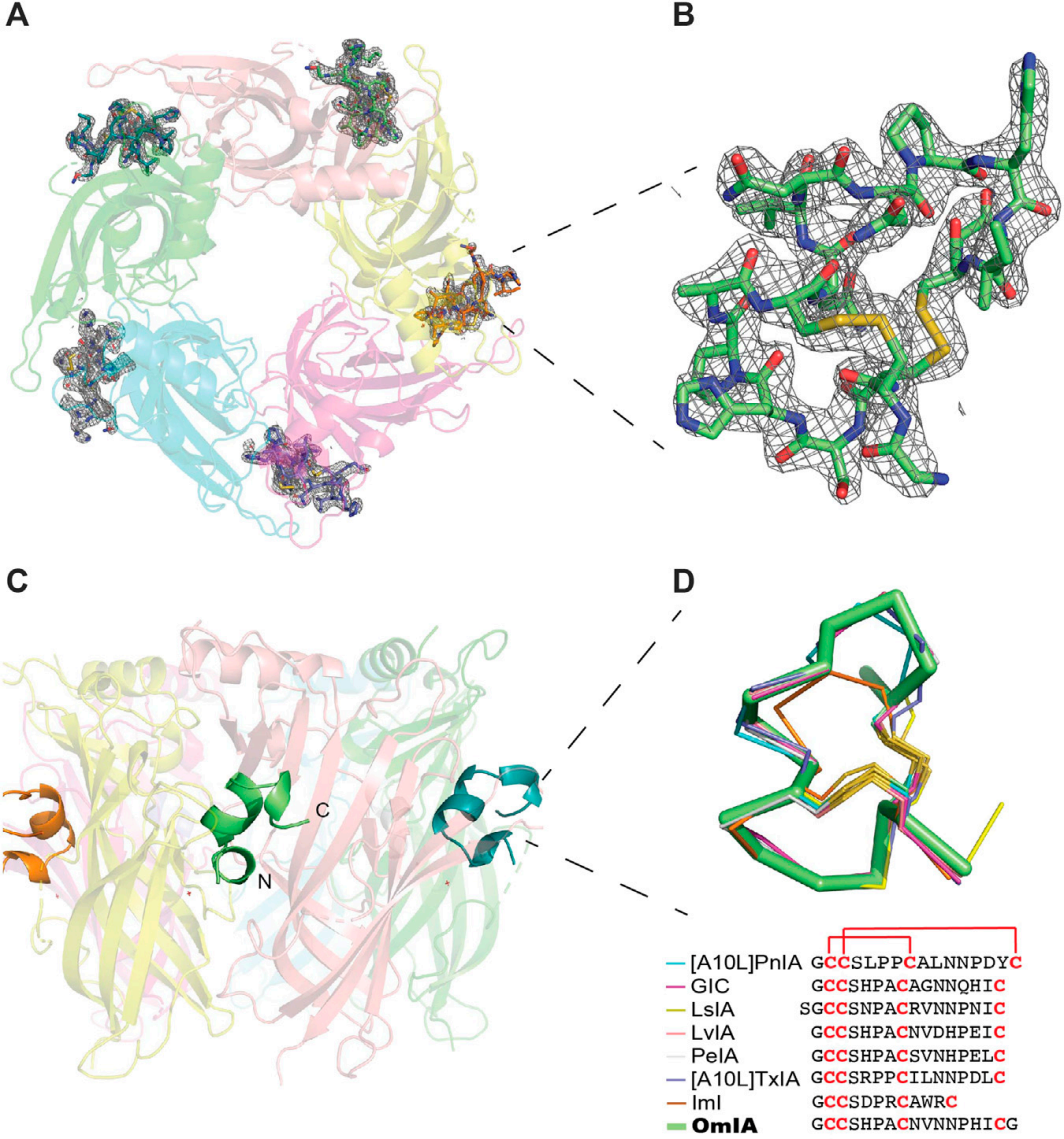
OmIA/*Ls*-AChBP co-crystal structure. **(A)** OmIA co-crystal structure with *Ls*-AChBP shows OmIA similarly residing in each of the five binding pockets. **(B)** OmIA the Fo-Fc map for the ligand contoured to 1.0 σ is also shown. **(C)** The typical binding mode of α-conotoxins is presented by OmIA in which its N and C termini orientate towards the top and bottom of the ligand binding pocket, respectively, while the central helix enters more deeply into the ligand binding pocket. **(D)** Superimposition of OmIA with previously co-crystallised α-conotoxins shows all bind with a similar backbone orientation [[A10L]PnIA PDB 2BR7, GIC PDB 5CO5, LsIA PDB 5T90, LvIA PDB 5XGL, PeIA PDB 5JME, [A10L]TxIA PDB 2UZ6, ImI PDB 2C9T].

A tight homopentameric ring assembly of subunits was observed for the OmIA/*Ls*-AChBP complex (Figure 1A), where OmIA occupied all five binding pockets located between two adjacent protomers. The N and C termini of bound OmIA orientated towards the top and bottom of the LBP, respectively, with the central helix abutting into the binding pocket. Structural superimposition with the HEPES*/Ls*-AChBP structure revealed that the C-loop of *Ls*-AChBP moved out a comparable distance (9.3 ± 0.4 Å from the Cys187 C_α_ atom) and had a similar backbone orientation to previously characterised co-crystal structures (Figure 1A). The backbone of bound co-crystallised OmIA also overlayed (RMSD = 1.53 ± 0.01 Å) the NMR solution structure of OmIA (PBD 2GCZ).

### Structural Basis for Interactions Between α-Conotoxin OmIA and *Ls*-AChBP

**FIGURE 2:**
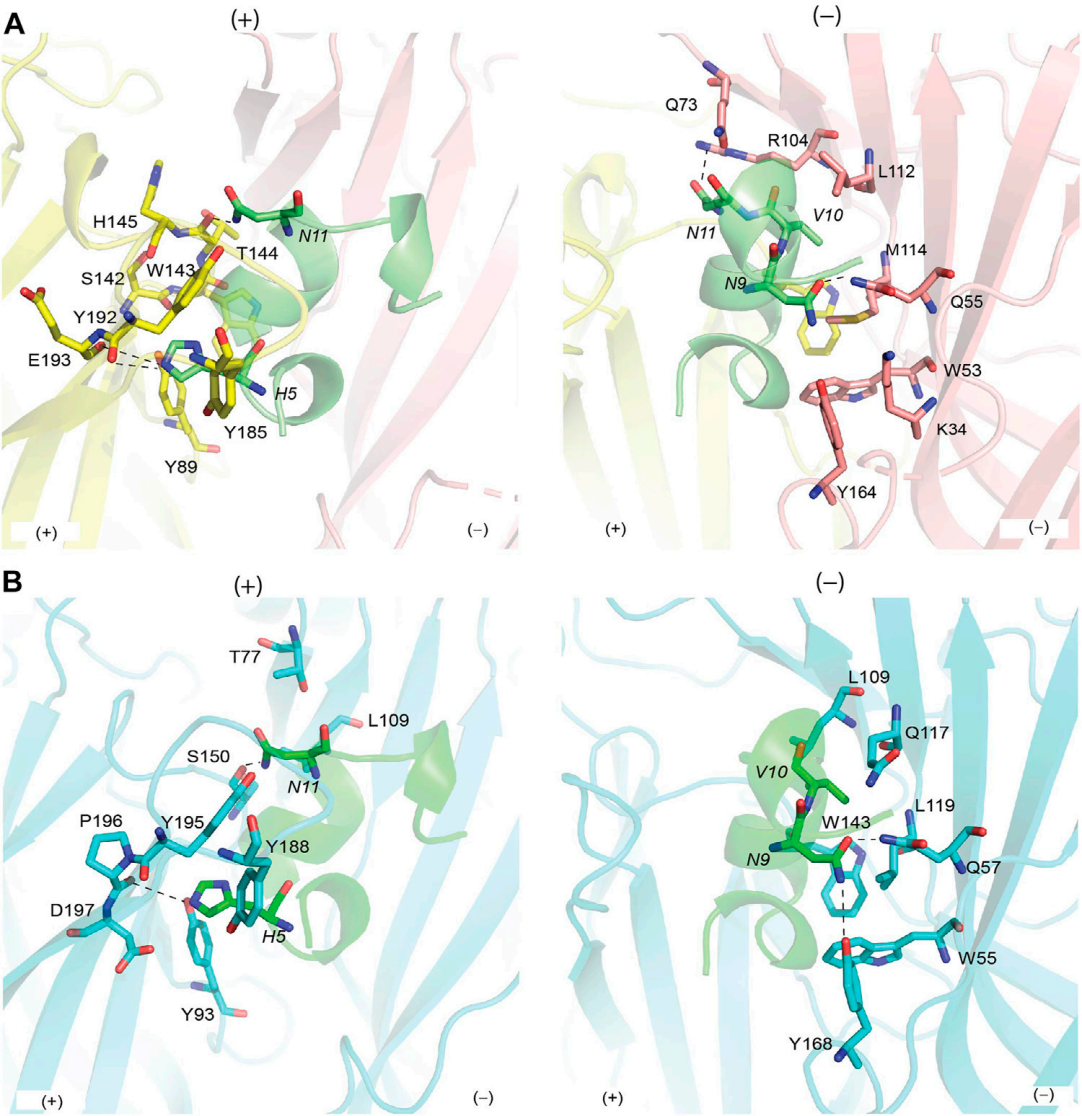
Binding interface between OmIA with Ls-AChBP **(A)** and homology model of   α7 nAChRs **(B)**. **(A)** His5 resides in the pocket consisting of conserved aromatic side-chain residues on the principal side of the *Ls*-AChBP, while hydrogen bond (dash line) is seen between Asn11 and backbone oxygen of Thr144. On the complementary side, Asn9 and Val10 show extensive contacts with the complementary side, with hydrogen bonds between Asn9/Gln55 and Asn9/Tyr164. (**B**) The binding surface of OmIA at  α7 nAChRs is comparable to *Ls*-AChBP. Hydrogen bonds between Asn9/Gln57 (equivalent to *Ls*-AChBP_Gln55), Asn9/Tyr167 (equivalent to *Ls*-AChBP_Tyr164), Asn11/backbone Ser14450 (equivalent to *Ls*-AChBP_Thr144) remain intact in the α7 subtype. Residues of OmIA are in italics.

Each of the five bound OmIA molecules interacted in a similar orientation (RMSD = 0.090 ± 0.005 Å) at the interface between two adjacent *Ls*-AChBP protomers that formed the principal and complementary binding sides (Figure 2A). Most interactions on the principal side were between His5 and Ala7 of OmIA and C-loop residues Tyr185–Tyr192, as well as interactions between the disulfide bridge of OmIA and the vicinal disulfide Cys188-Cys189 of *Ls*-AChBP. His5 resided in a pocket formed by aromatic side-chain residues, specifically Tyr89, Tyr192 and Tyr185, and formed hydrogen bond with Tyr89 and the backbone oxygen of Glu193. Ala7 interacted with Ser142, Trp143, Thr144, His145 and Tyr192. On the complementary side, OmIA_Asn9 interacted with a surface comprising charged residue Lys34, polar residues Ser32, Gln55, Tyr164, and hydrophobic residue Met114, in which hydrogen bonds were seen between Asn9/Gln55 and Asn9/Tyr164 (Figure 2A). OmIA_Val10 interacted in a pocket formed by Thr144 on the principal side and Arg104, Leu112 and Met114 on the complementary side of *Ls*-AChBP. The surface interacting with OmIA_Asn11 in the co-crystal structure comprised mostly polar and charged residues from both the principal (Thr144, Glu149 and Tyr192) and complementary faces of each subunit interface (Gln73 and Arg104), where hydrogen backbone were seen between Asn11/Thr144 and Asn11/Gln73 (Figure 2A). Interactions between OmIA and *Ls*-AChBP are summarized in Supplementary Table S2.

### Homology Model of OmIA Bound to α7 nAChRs

OmIA potently blocks human α7 nAChRs with an IC_50_ of 27 nM (Talley et al., 2006). Using the co-crystal structure of *Ls*-AChBP/OmIA as a template, a homology model of OmIA bound to α7 nAChRs was constructed (sequence alignment reported in Supplementary Figure S1). The OmIA/α7 complex revealed OmIA interacted similarly at α7 nAChR and *Ls*-AChBP. At the principal face, His5 formed π–π interactions with the conserved aromatic side chains of Tyr93, Tyr188 and Tyr195, as well as an interaction with negatively charged Asp197. A hydrogen bond between His5 and the backbone oxygen of α7_Pro196 (equivalent to *Ls*-AChBP_Glu193) observed in the co-crystal structure also contributed to the docking pose at human α7 nAChRs (Figure 2B). On the complementary face, OmIA_Asn9 interacted with polar Gln57, hydrophobic Trp55, Tyr168, and Leu119 in the α7 subtype, similar to the interactions observed at *Ls*-AChBP. OmIA_Val10 interacted with Trp149 on the principal side and a hydrophobic triad comprising Leu109, Gln117 and Leu119 on the complementary side of the α7 nAChRs. OmIA_Asn11 interacted with a more hydrophobic surface comprising Ser150 on principal side and Thr77 and Leu109 on complementary side (Figure 2B). Based on these interactions, we synthesized [H5R]OmIA to evaluate whether the insertion of positively charged residue would disrupt the π–π interactions. Additionally, less favourable interactions with residues on the complementary side of α7 nAChRs were introduced in [N9H]OmIA, [V10Q]OmIA and [N11D]OmIA to assess their potential to disrupt contacts with α7 nAChRs (Supplementary Table S3). The CD spectroscopy profile for OmIA and analogues were consistent with that expected for an α–helical structure (Supplementary Figure S2).

### Functional Characterisation of OmIA Analogues at *Ls*-AChBP and α7 nAChRs in the Presence of PNU120596

**FIGURE 3:**
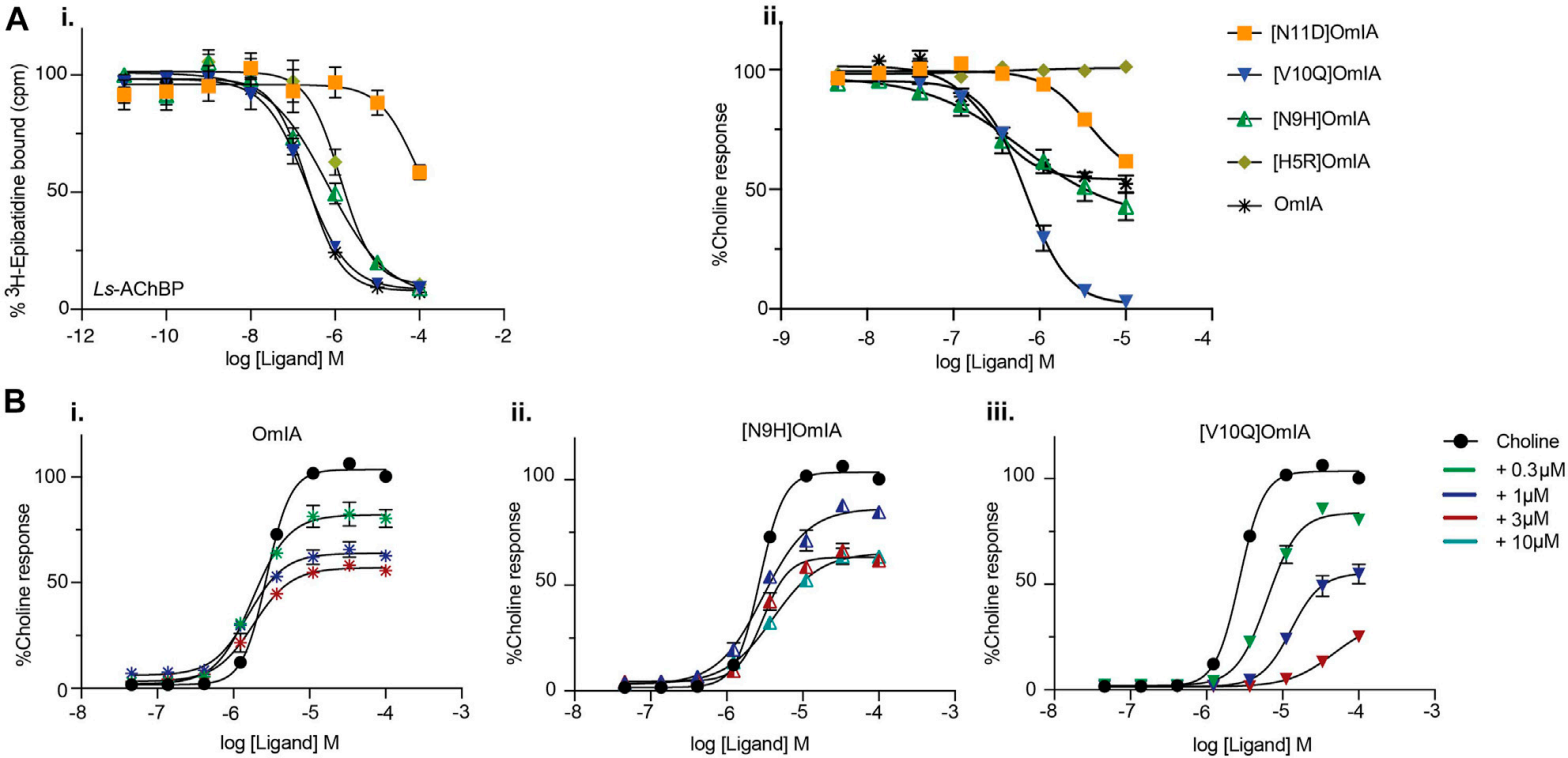
Activity of OmIA and its analogues on Ls-AChBP **(A)** and α7 nAChRs with the addition of nAChR type II PAM PNU120596 **(B)**. **(A)** Displacement of (^3^H)-epibatidine from *Ls*-AChBP (i) and the inhibition of choline activation of α7 nAChRs measured in a FLIPR Ca^2+^ influx assay on SH-SY5Y cells with the addition of nAChR type II PAM PNU-120596 (ii) by OmIA and its analogues. **(B)** Concentration-response curves for choline alone (black round) and in the presence of increasing concentrations of OmIA (i), [N9H]OmIA (ii), and [V10Q]OmIA (iii). Data represent mean ± SEM of triplicate data from three independent experiments.

To validate the observations from the co-crystal structure of OmIA/*Ls*-AChBP, the binding of OmIA analogues were first examined on *Ls*-AChBP. [H5R]OmIA, [N9H]OmIA and [N11D]OmIA showed 3.6-, 2.6- and 10-fold reduced affinity for *Ls*-AChBP respectively, while the ability of [V10Q]OmIA to displace [^3^H]-epibatidine at *Ls*-AChBP was unaffected, compared to OmIA (Figure 3Ai and Table 1). In contrast to results from binding studies, OmIA and [N9H]OmIA only partially (∼ 50%) inhibited α7 nAChR responses to choline in SH-SY5Y cells, with 95% CI of the curve bottoms not overlapping 0%, while [V10Q]OmIA was a near full inhibitor in the presence of the type II PAM, PNU120596 (Figure 3A(ii) and Table 1). The inhibitory activity of all analogues decreased at α7 nAChRs, with [H5R]OmIA and [N11D]OmIA experiencing the largest drop in potency. Using a pairwise comparison of the IC_50_ values for binding affinity for *Ls*-AChBP and potency at human α7 nAChRs, all analogues were significantly (*p* < 0.05) different to wildtype OmIA.

**TABLE 1:** IC_50_ values for displacement of (^3^H)-epibatidine binding on *Ls*-AChBPs and the inhibition of choline activation at α7 nAChR in SH-SY5Y cells in the presence of PNU120596 by OmIA and its analogues. *Ratios were calculated between OmIA and its analogues. Data represent mean ± SEM of triplicate data from three independent experiments. ^a^denotes significant difference in IC_50_ values to wildtype OmIA (*p* < 0.05). ^b^denotes 95% CIs for curve bottom values non-overlapping 0%.

	[3H]-epibatidine binding, IC_50_ ± SEM (μM)		FLIPR SH-SY5Y, IC_50_ ± SEM (μM)	
	*Ls*-AChBP	Ratio*	α7 nAChRs	Ratio*
OmIA	0.28 ± 0.07	1	0.27 ± 0.02	1^b^
[H5R]OmIA	1.00 ± 0.04	3.57^a^	>10	>10^a^
[N9H]OmIA	0.73 ± 0.05	2.61^a^	0.71 ± 0.19	2.63^a,b^
[V10Q]OmIA	0.22 ± 0.05	1	0.72 ± 0.10	2.67^a^
[N11D]OmIA	>10	>10^a^	3.80 ± 0.06	14.07^a^

To further characterise the partial inhibition observed in SH-SY5Y cells, a fixed concentration of OmIA and its analogues was preincubated with PNU120596 prior to the addition of increasing concentrations of choline. A decrease of the agonist’s maximal response, where 95%CI of the curve top values did not overlap 100%, without a significant change in the agonist EC_50_ (*p* > 0.05), was observed for OmIA and [N9H]OmIA. The degree of maximal response depression increased as the concentration of OmIA and analogues increased, suggesting insurmountable antagonism by OmIA. In contrast, [V10Q]OmIA depressed the maximal response and caused a rightward shift in the EC_50_ for choline (*p* < 0.05), suggesting [V10Q]OmIA has a different mechanism of action compared to OmIA and [N9H]OmIA (Figure 3B and Table 2).

**TABLE 2:** Inhibition of choline activation of α7 nAChRs in SH-SY5Y cells by OmIA and analogues in the presence of PNU120596. Data represent mean ± SEM of triplicate data from three independent experiments. ^b^denotes 95% CIs for curve top values non-overlapping 100%.^c^ denotes significant difference in EC_50_ values to agonist alone (*p* < 0.05).

Peptide concentration		Choline EC_50_ ± SEM (μM)
Choline		3 ± 0.26
OmIA	0.3 μM	2 ± 0.28^b^
	1 μM	2 ± 0.32^b^
	3 μM	2 ± 0.63^b^
[N9H]OmIA	1 μM	3 ± 0.52^b^
	3 μM	3 ± 0.36^b^
	10 μM	5 ± 1.20^b^
[V10Q]OmIA	0.3 μM	6 ± 0.65
	1 μM	13 ± 0.44^b,c^
	3 μM	45 ± 4.80^b,c^

### Does Partial Inhibition by OmIA and Analogues Require PNU-120596?

**FIGURE 4:**
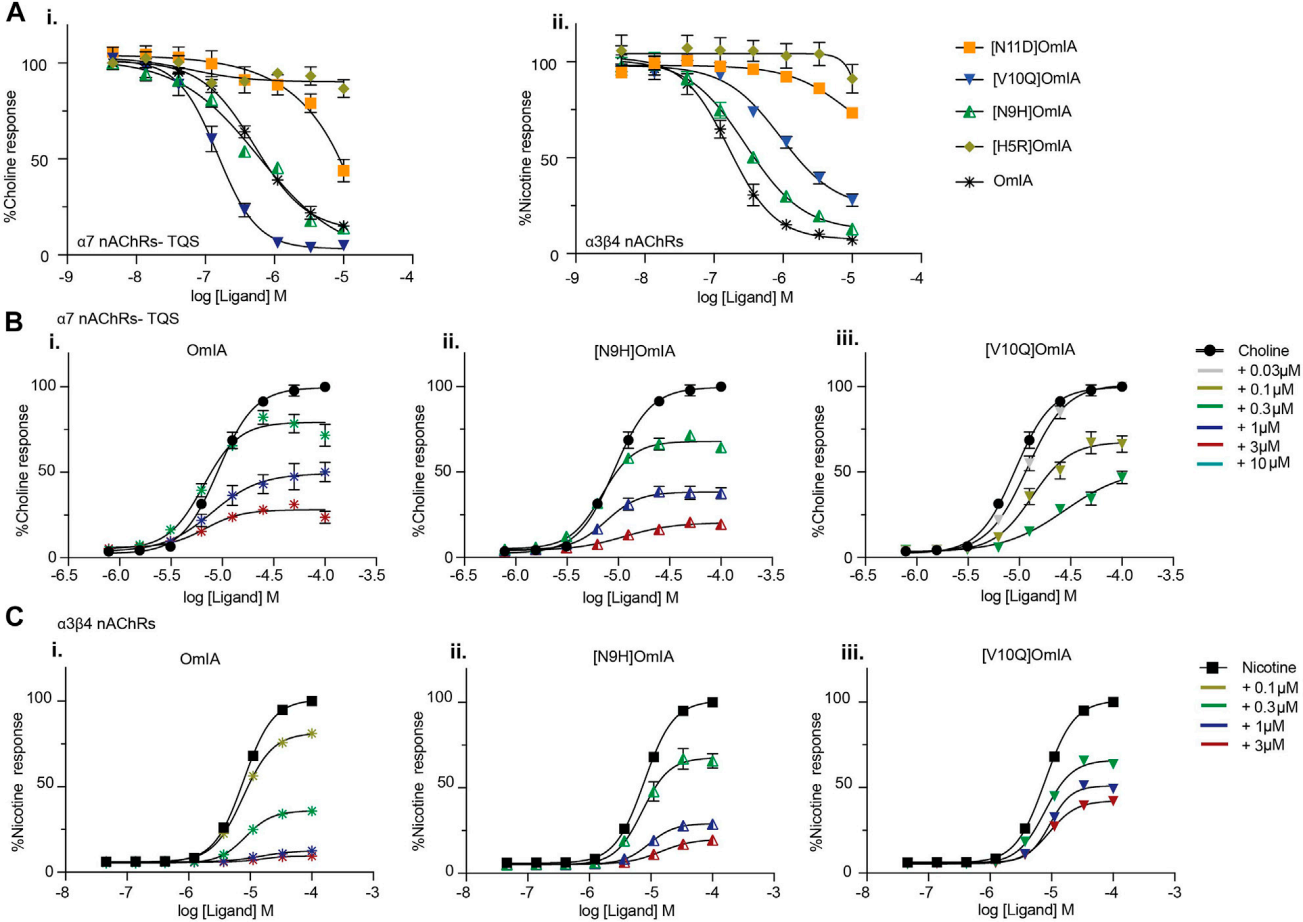
Activity of OmIA and its analogues at α7 nAChRs in the presence of the type II PAM TQS, and at α3β4 nAChRs. **(A)** Inhibition of choline activation of α7 nAChR in the presence of TQS (i) and inhibition of nicotine activation of α3β4 nAChRs (ii) by OmIA and analogues using FLIPR Ca^2+^ influx assay results in SH-SY5Y cells Concentration-response curves for nicotine (filled square) and choline (filled circle) in the presence of varying concentrations of OmIA (i), [N9H]OmIA (ii) and [V10Q]OmIA (iii) at TQS-α7 nAChRs **(B)** and at α3β4 nAChRs **(C)**. Data represent mean ± SEM of triplicate data from three independent experiments.

To examine whether the partial inhibition produced by OmIA and analogues at α7 nAChRs only occurs in the presence of PNU120596, we examined their effects on α3β4 nAChR responses, and α7 nAChR responses in the presence of a different type II PAM, TQS, acting like PNU120596 to stabilize the open state of the α7 nAChRs (Halvard Grønlien et al., 2007). Interestingly, at both nAChR subtypes, at the highest concentration tested, OmIA and [N9H]OmiA had significant residual responses (∼ 15%) (95% CI did not overlap 0%), albeit to a lesser extent than observed with PNU120596, except for (V10Q) OmIA (Figure 4A). While [N9H]OmIA retained wild-type potency, both [H5R]OmIA and [N11D]OmIA had reduced IC_50_ (*p* < 0.05), and [V10Q]OmIA had 3.6-fold increased potency at α3β4 nAChRs and 6-fold decreased potency at α7 nAChRs in the presence of TQS compared to wildtype (*p* < 0.05) (Figure 4A and Table 3). Similar to observations at α7 nAChRs in the presence of PNU120596, maximal α3β4 and TQS-α7 nAChR responses were significantly depressed without altering the EC_50_ for choline, except [V10Q]OmIA also showed an increased EC_50_ for choline at nAChRs in the presence of TQS (*p* < 0.05) (Figures 4B,C and Table 4).

**TABLE 3:** IC_50_ values for the inhibition of choline activation of α7 nAChRs with the addition of nAChR type II PAM TQS and α3β4 nAChRs in SH-SY5Y cells by OmIA and its analogues. *Ratios were calculated between OmIA and its analogues. Data represent mean ± SEM of triplicate data from three independent experiments. ^a^denotes significant difference in IC_50_ values to wildtype OmIA (*p* < 0.05). ^b^denotes 95% CIs for curve bottom values non-overlapping 0%.

	FLIPR SH-SY5Y, IC_50_ ± SEM (μM)			
	TQS-α7 nAChRs (μM)	Ratio*	α3β4 nAChRs (μM)	Ratio*
OmIA	0.50 ± 0.09	1^b^	0.16 ± 0.03	1 ^b^
[H5R]OmIA	>10	>10^a^	>10	>10
[N9H]OmIA	0.54 ± 0.09	1^b^	0.29 ± 0.04	1.69^b^
[V10Q]OmIA	0.14 ± 0.03	0.28^a^	0.92 ± 0.16	5.75^a,b^
[N11D]OmIA	>10	>10	>10	>10

**TABLE 4:** Effect of OmIA and its analogues on choline concentration-activation curve at α7 nAChRs with the addition of nAChR type II PAM, TQS and on nicotine concentration-activation curve at α3β4 nAChRs in SH-SY5Y cells. ^b^denotes 95% CIs for curve top values non-overlapping 100%. ^c^denotes significant difference in EC_50_ values to agonist alone (*p* < 0.05). Data represent mean ± SEM of triplicate data from three independent experiments.

	Peptide concentration	Choline EC_50_ ± SEM (μM) at TQS-α7 nAChRs	Nicotine EC_50_ ± SEM (μM) at α3β4 nAChRs
Choline		9 ± 0.90	
Nicotine			8 ± 0.35
OmIA	0.1 μM		8 ± 1.40^b^
	0.3 μM	7 ± 0.62^b^	8 ± 0.50^b^
	1 μM	7 ± 0.29^b^	13 ± 3.40^b^
	3 μM	7 ± 0.53^b^	
[N9H]OmIA	0.3 μM	7 ± 0.12^b^	10 ± 0.67^b^
	1 μM	8 ± 0.72^b^	10 ± 1.50^b^
	3 μM	11 ± 1.10^b^	13 ± 0.23^b^
	10 μM		
[V10Q]OmIA	0.03 μM	12 ± 1.80	
	0.1 μM	14 ± 0.86^b,c^	
	0.3 μM	32 ± 11.70^b,c^	8 ± 0.74^b^
	1 μM		9 ± 0.39^b^
	3 μM		9 ± 1.10^b^

### Long Side Chain Amino Acids at Position 10 Differentially Effect OmIA Pharmacology

**FIGURE 5:**
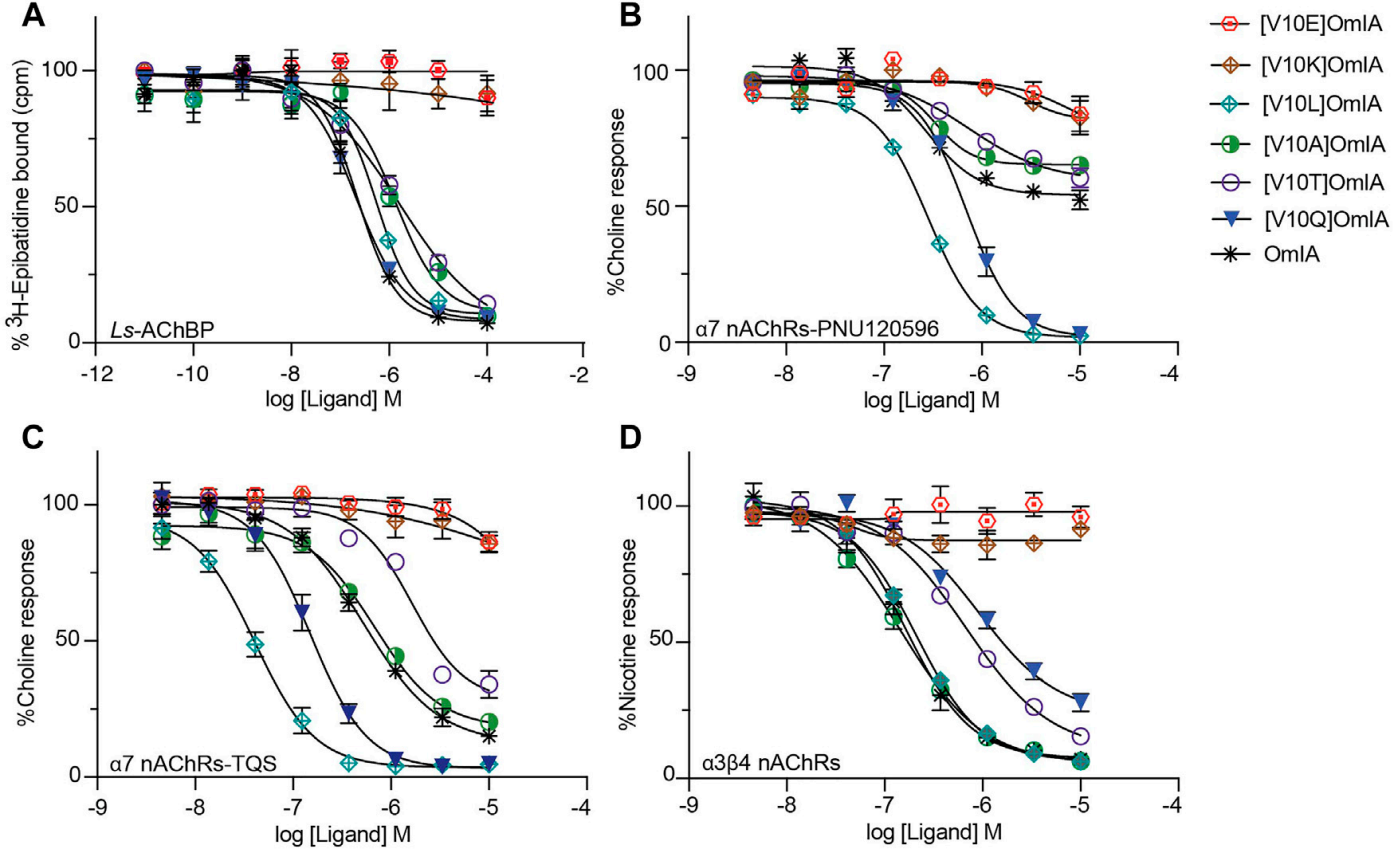
Activity of OmIA and its analogues at position 10 at Ls-AChBP, α7 nAChRs with the addition of nAChR type II PAM PNU-120596, TQS and α3β4 nAChRs. **(A)** Displacement of [^3^H]-epibatidine from *Ls*-AChBP. The inhibition of choline activation of α7 nAChRs with PNU-120596 **(B)**, TQS as PAMs **(C)** and inhibition of nicotine activation of α3β4 nAChRs **(D)** by OmIA and its analogues at position 10, measured in a FLIPR Ca^2+^ influx assay on SH-SY5Y cells. Data represent mean ± SEM of triplicate data from three independent experiments.

Given the differential pharmacological effects of [V10Q]OmIA compared to wildtype and other analogues, amino acids with different chemical properties were substituted at position 10 of OmIA, specifically [V10E]OmIA, [V10K]OmIA, [V10L]OmIA and [V10A]OmIA to examine the role of residue 10 in modulating OmIA pharmacology (Supplementary Table S3). The substitutions at position 10 made no change to the secondary structure compared to OmIA and decreased OmIA binding affinity at *Ls*-AChBP, with the largest reduction exhibited by [V10K]OmIA and [V10E]OmIA (*p* < 0.05, except for [V10L]OmIA (Supplementary Figure S2, Figure 5A and Supplementary Table S4).

At nAChRs, the substitution of long charged side chain amino acid into position 10 abolished OmIA activity (*p* < 0.05) (Figures 5B–D and Supplementary Table S4). Meanwhile, at the highest concentration tested, [V10A]OmIA and [V10T]OmIA displayed residual response at both nAChR subtypes (95%CI nonoverlapping with 0%), with the most significant effect exhibited by [V10A]OmIA (65%) and [V10T]OmIA (60%) at PNU120596-α7 nAChRs (Figure 5B). While [V10A]OmIA retained its potency, [V10T]OmIA showed a decrease in activity with the highest loss of 10-fold observed at TQS-α7 nAChRs (*p* < 0.05) (Figure 5C). Replacement of Val10 in OmIA with a long hydrophobic amino acid (Leu) increased OmIA IC_50_ 10-fold at TQS-α7 nAChRs (*p* < 0.05) but slightly decreased OmIA activity at α3β4 nAChRs (*p* > 0.05).

**FIGURE 6:**
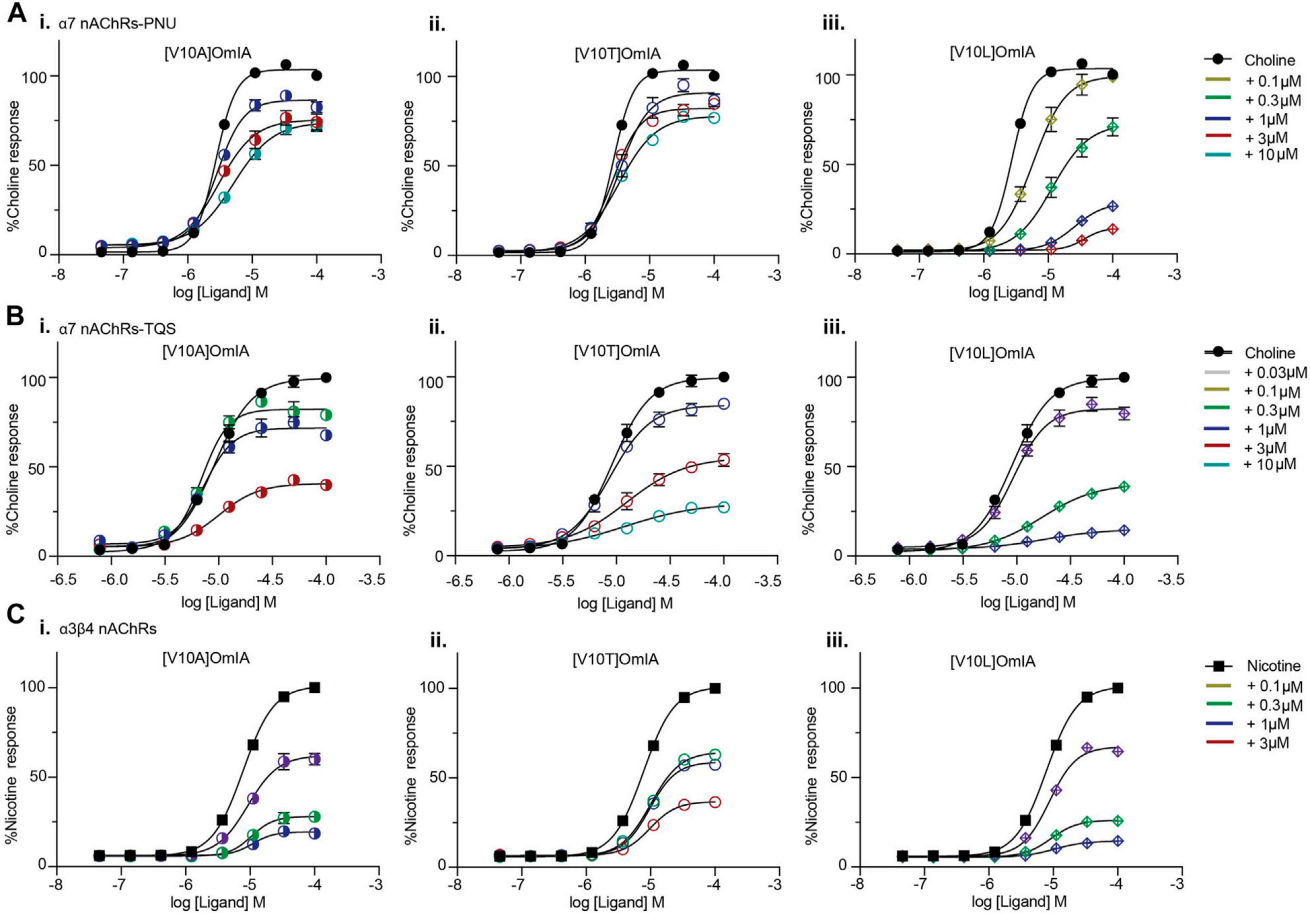
Concentration-response curves for nicotine (black square) alone, choline alone (black round) in the presence of OmIA analogues at position 10 at α7 nAChRs with the addition of nAChR type II PAM, PNU120596 **(A)**, TQS **(B)** and at α3β4 nAChRs **(C)**. Data represent mean ± SEM of triplicate data from three independent experiments.

All active OmIA analogues at position 10 also depressed the maximal response of agonist-activation curves at both nAChR subtypes significantly (Supplementary Table S5 and Figure 6). However, similar to [V10Q]OmIA, only [V10L]OmIA both decreased PAMs-α7 maximal response and increased the EC_50_ of choline (*p* < 0.05).

### OmIA and Analogues Displayed Biphasic Concentration-Inhibition Curves at PNU120596-α7 nAChRs

**FIGURE 7:**
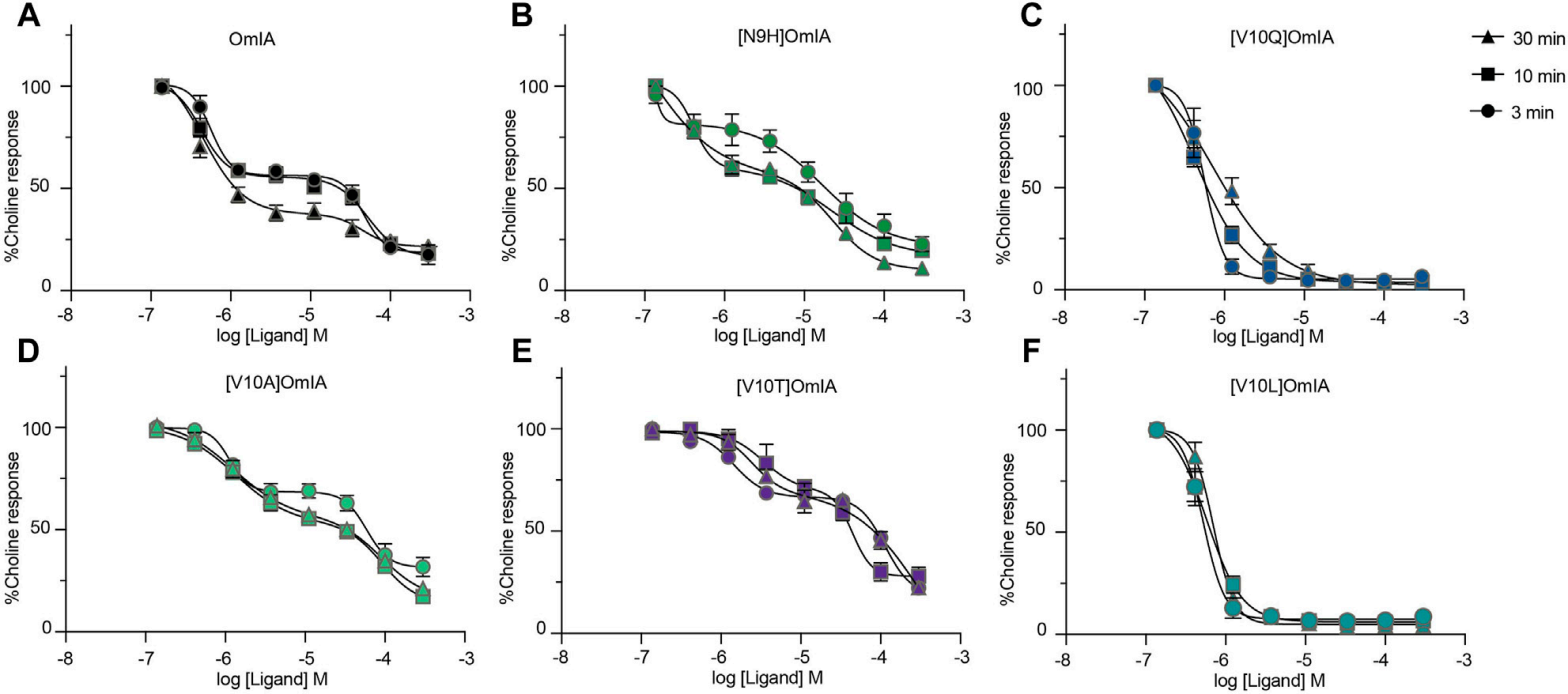
OmIA and selected analogues show biphasic inhibition of α7 nAChRs in the presence of a type II PAM (PNU120596) pre-incubated at 3, 10 or 30 min. Data represent mean ± SEM of triplicate data from three independent experiments.

To further characterise the partial inhibition of OmIA and its analogues at PNU120596-α7 nAChRs, we compared full inhibitor dose-response curves at three incubation periods. Intriguingly, the dose-response relationship to OmIA and other analogues, except for [V10Q]OmIA and [V10L]OmIA, showed biphasic inhibition with clear high and low affinity binding sites (Figure 7 and Table 5). With short preincubations, OmIA exhibits equal ratio between the high-affinity and low affinity site, while [N9H]OmIA, [V10A]OmIA and [V10T]OmIA preferred to bind to the low-affinity binding site. As the preincubation period increased from 3 to 30 min, there was an associated increase in the high affinity fraction (Table 5). OmIA, [N9H]OmIA and [V10Q]OmIA displayed a comparable antagonism to wildtype OmIA, while [V10A]OmIA and [V10T]OmIA showed the highest loss in potency (*p* < 0.05) (Figure 7 and Table 5).

**TABLE 5:** The IC_50_ (µM) of OmIA and analogues following inhibitor preincubations of 3, 10 or 30 min at PNU120596-modified α7 nAChRs. Data represent mean ± SEM of triplicate data from three independent experiments.

		OmIA	(N9H)OmIA	(V10Q)OmIA	(V10A)OmIA	(V10T)OmIA	(V10L)OmIA
IC_50__1 ± SEM	3 min	0.41 ± 0.04	0.24 ± 0.01	0.39 ± 0.11	1.1 ± 0.0^a^	1.7 ± 0.46^a^	0.47 ± 0.03
	10 min	0.61 ± 0.16	0.41 ± 0.01	0.50 ± 0.10	1.6 ± 0.77^a^	1.7 ± 0.17^a^	0.62 ± 0.11
	30 min	0.38 ± 0.06	0.41 ± 0.0	0.50 ± 0.19	1.5 ± 0.39^a^	2.8 ± 0.42^a^	0.69 ± 0.04
IC_50__2 ± SEM	3 min	50.0 ± 9.0	30.0 ± 7.0	NA	80.0 ± 3.0^a^	40.0 ± 1.0^a^	NA
	10 min	50.0 ± 1.0	20.0 ± 3.3^a^	NA	90.0 ± 1.0^a^	100.0 ± 3.0^a^	NA
	30 min	50.0 ± 4.1	10.0 ± 3.1^a^	NA	80.0 ± 16.0^a^	120.0 ± 3.5^a^	NA
Fraction 2 ± SEM	3 min	0.60 ± 0.02	0.39 ± 0.09	NA	0.37 ± 0.04	0.31 ± 0.05	NA
	10 min	0.51 ± 0.02	0.42 ± 0.02	NA	0.58 ± 0.04	0.46 ± 0.06	NA
	30 min	0.72 ± 0.02	0.54 ± 0.02	NA	0.57 ± 0.11	0.51 ± 0.02	NA

a Denotes significant difference in IC_50_ values to wildtype OmIA (*p* < 0.05).

## DISCUSSION

α-Conotoxins constitute the largest group of characterized *Conus* peptides that target nAChRs with high potency and selectivity and have contributed significantly to our understanding of nAChR pharmacology (Lewis and Garcia, 2003; Lewis et al., 2012). OmIA isolated from *Conus omaria* venom is an α4/7-conotoxin that exhibits high potency at α7 and α3β2 nAChRs. In this study, we present the co-crystal structure of OmIA with *Ls*-AChBP, and a new homology model of OmIA bound at α7 nAChRs, which revealed His5, Val10 and Asn11 were key contributors to OmIA binding at α7 nAChRs. Interestingly, OmIA and most analogues acted as functional insurmountable antagonists, while those with long side chain at position 10 were partial surmountable inhibitors at α7 nAChRs in the presence of type II PAMs. OmIA and analogues also displayed biphasic inhibition at α7 nAChRs in the presence of PNU120596.

The co-crystal of OmIA with *Ls*-AChBP revealed a similar binding orientation to other α-conotoxins, including several overlapping pairwise interactions with these α-conotoxins (Figure 1D). However, differences in the residues interacting at the binding sites were also observed that likely underlie differences in α-conotoxin pharmacology and selectivity towards distinct nAChR subtypes (Celie et al., 2005; Hansen et al., 2005; Dutertre et al., 2007; Lin et al., 2016; Xu et al., 2017). From the co-crystal structure of OmIA/*Ls*-AChBP and OmIA docking to α7 nAChRs, we identified His5, Val10 and Asn11 as potential key residues for high potency at α7 nAChRs. In support, [H5R]OmIA showed a significant drop in potency, possibly arising from the disruption of the π-π interaction with Tyr93, Tyr188, Tyr195 as well as the loss in hydrogen bond between His5 and α7_Pro196 backbone (Figure 2B). Despite similarities in the interacting surface of OmIA, [A10L]TxIA, [A10L]PnIA and GIC (Celie et al., 2005; Dutertre et al., 2007; Lin et al., 2016), the H5R mutation in [L5R A10L]TxIA had no influence on potency at α7 nAChRs, while [H5A]GIC lost all activity α7 nAChRs, indicating that the role played by position 5 is highly variable across the different α-conotoxins. In the co-crystal structure, Val10_ OmIA was seen to occupy the previously characterised hydrophobic funnel Trp143 on the principal face and Leu112, Met114 on the complementary face of human α7 nAChRs that favoured interactions with hydrophobic residues (Celie et al., 2005; Dutertre et al., 2007; Hopping et al., 2014). Interestingly, while at PNU120596-α7 nAChRs the [V10Q]OmIA potency decreased but [V10L]OmIA potency remained unchanged, at TQS-α7 nAChRs, both the [V10L]OmIA and [V10Q]OmIA potency increased, suggesting differential effects can be modulated by different PAMs. Finally, [N11D]OmIA introduced a likely clash with the complementary hydrophobic interacting surface of α7 nAChRs, causing a significant drop in potency (Supplementary Figure S2). Interestingly, [N9H]OmIA introduced differential effects at α7 nAChRs in the presence of different PAMs, suggesting a novel role for position 9 in modulating OmIA activity (Supplementary Figure S2). Previously, position 9 was identified as important for the potency of a range of α-conotoxins (Halai et al., 2009; Azam et al., 2010; Hone et al., 2013; Hone et al., 2019). These studies suggest that further characterisation of the role played by these key positions in OmIA is warranted.

**FIGURE 8:**
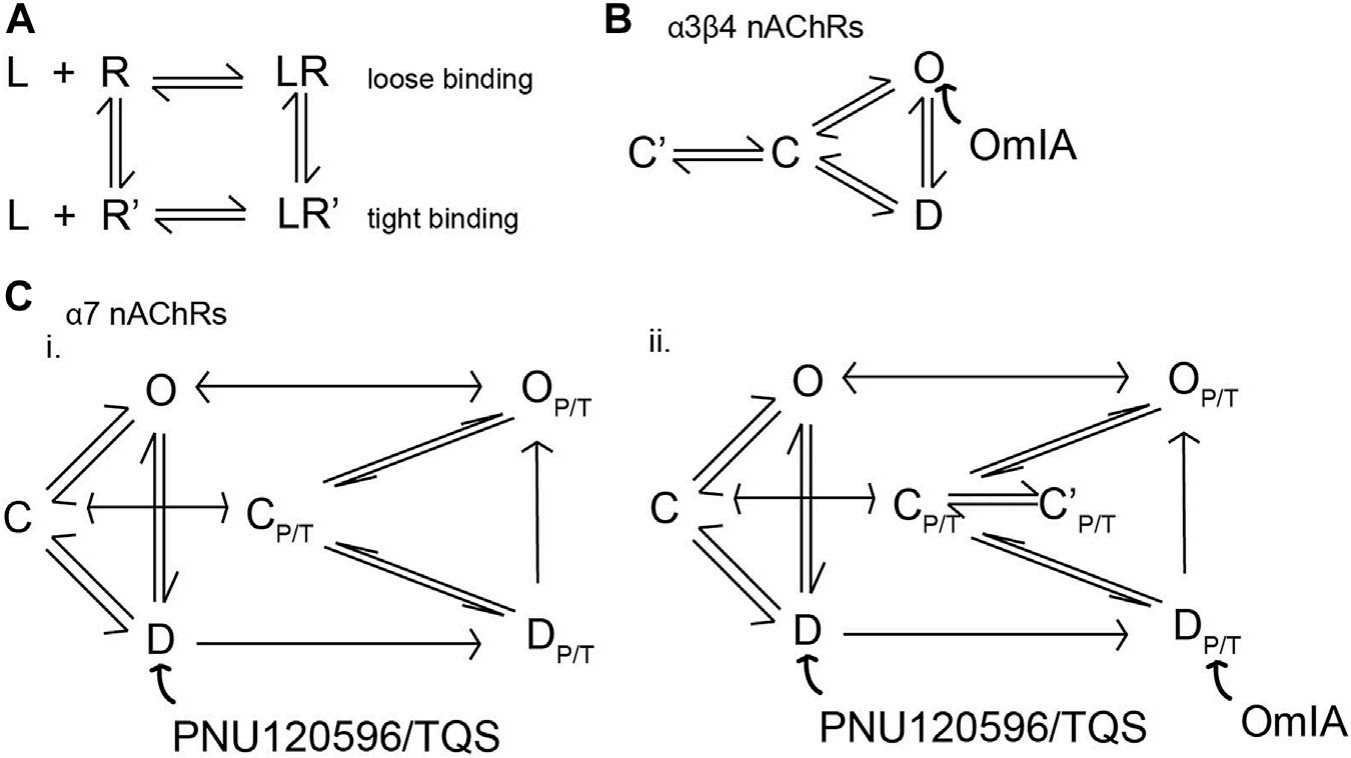
Proposed model depicting conformational changes of nAChRs induced by the binding of OmIA. **(A)** Schematic representation of the potential mechanism of potential responsible for surmountable and insurmountable antagonism. When antagonists (L) bind to receptors (R), the antagonists-receptors may adopt two states: LR, a loose binding state accounting for the surmountable inhibition by the antagonists, and LR’, a tight binding state accounting for the insurmountable inhibition by the antagonist. **(B)** The model of the activation of α3β4 nAChRs with three basic conformational states, resting or closed (C), inactivated or desensitized (D) and conducting or open state (O). OmIA bound to resting-state of nAChR, converting α3β4 nAChRs into close state. The OmIA-receptor complex may then convert into a tight binding state (C’) that are slowly reversible and cannot be overcome during the short exposure of agonist, meaning that only part of the receptors can be liberated, and hence occupied and stimulated by the subsequence addition of agonist. This feature accounts for the insurmountable antagonism of OmIA and its analogues. **(C)** The PNU120596/TQS-bound channel exists in parallel (C_P/T_, D_P/T_, O_P/T_) states (i). PNU120596/TQS predominantly binds to the desensitized state (D) that transforms the channel to a PNU120596/TQS-modified channels (D_P/T_) which is energetically favourable to convert to a PNU120596/TQS-modified Open (O_P/T_) (Szabo et al., 2014). OmIA and its (ii) binding to the D_P/T_ and convert the receptor to the closed (C_P/T_) state. Here, OmIA, [N9H]OmIA, [V10A]OmIA and [V10T]OmIA predominantly exists in the tight binding complex (C’_P/T_), accounting for its fully surmountable inhibition. Meanwhile, [V10Q]OmIA and [V10L]OmIA complexes with receptors coexist in the loose binding state (C_P/T_) and the tight binding state (C’_P/T_), accounting for its partial surmountable inhibition.

Surprisingly, OmIA and selected analogues displayed insurmountable antagonism of functional nAChR responses at α7 nAChRs in the presence of type II PAMs and at α3β4 nAChRs. Insurmountable antagonism may arise through allosteric inhibition (Kenakin, 2017); however, OmIA binds at the orthosteric sites, consistent with a competitive interaction with the orthosteric agonists investigated. Alternatively, OmIA might stabilise nAChRs in a desensitized or a desensitized-like state without transitioning through the open state, resulting in decreased responsiveness of the receptor for a subsequent stimulus by agonists (Hurst et al., 2013). Antagonist stabilizing the desensitized state rather than the closed state of nAChRs was previously proposed for the competitive nAChR antagonist dihydro-β-erythroidine (DH*β*E) (Bertrand et al., 1992). In agreement with this proposal, the co-crystal complex of DH*β*E/AChBP revealed that DH*β*E induced closing of the nAChR_C-loop typical of agonist binding, and established a hydrogen-bonding network similar to agonists (Shahsavar et al., 2012). However, these features were not observed in the OmIA/*Ls*-AChBP complex, suggesting OmIA is unlikely to stabilise a desensitized state. Insurmountable antagonism could also be due to the presence of different receptor subpopulations (Kenakin, 2017). Despite SH-SY5Y cells expressing the β2 nAChR subunit, the presence of a novel heteromeric α7β2 nAChRs is not well documented. However, the possibility of this subpopulation potentially activated by choline analogues or subpopulations of α7 nAChRs in pharmacologically distinct activatable states that are not blocked by OmIA cannot be discounted (Vetter and Lewis, 2010) until effects on oocytes expressed homomeric α7 nAChRs and heteromeric α7β2 nAChRs are compared. Alternatively, the insurmountable action of OmIA may arise from non-equilibration interactions between antagonist and agonist since peak agonist responses are rapidly and transiently induced, while antagonist responses are slower to reverse (Vauquelin et al., 2001; Vauquelin et al., 2002; Charlton and Vauquelin, 2010; Kenakin, 2017). This effect could also be interpreted as “pseudo-irreversible” antagonism, as the antagonist-receptor complex could be considered irreversible within the time-frame of the agonist responses (Kenakin, 2017). Remarkably, [V10Q]OmIA and [V10L]OmIA not only displayed a depression in maximal response but also induced a right-ward shift to the concentration-response of choline at PNU120596/TQS-α7 nAChRs, characteristic of surmountable inhibition (Charlton and Vauquelin, 2010). This phenomenon could reflect the ability of the antagonist-receptor complexes to adopt two distinct states, a fast reversible state (for the surmountable inhibition) and a slowly reversing binding state (for insurmountable inhibition). In a “two-state, two-step” model explaining these observations (Figure 8), the antagonist (L) binds to the receptor to form a loose binding complex (L.R) which can convert into a tight binding complex (L.R*). The antagonist is fully surmountable if L.R predominates, partially insurmountable when L.R and L.R* coexist and fully insurmountable when all complexes are in the L.R* state (Kenakin, 1993; Vauquelin et al., 2001). Apparently, OmIA, [N9H]OmIA, [V10A]OmIA and [V10T]OmIA bind to PNU120596-α7 nAChRs in a tight binding state, resulting in fully insurmountable antagonism. In contrast, [V10L]OmIA and [V10Q]OmIA interact in both the LR (loose form) and LR* (tight form) states. Upon stimulation by agonist, a greater percentage of the pre-formed antagonist receptor complex could be free to be occupied by agonists, explaining for the rightward shift of agonist EC_50_ (Figure 8). Alternatively, [V10Q]OmIA and [V10L]OmIA might have faster off-rate that would allow a component of binding to be competitively inhibited.

Another unique feature identified in these studies is the ability of OmIA and [N9H]OmIA to display high affinity partial inhibition and a lower affinity inhibitory action at PNU120596-α7 nAChRs. Partial inhibition by OmIA and [N9H]OmIA may arise through OmIA being a partial agonist, however, OmIA and all analogues were unable to induce Ca^2+^ current (Supplementary Figure S3), or because OmIA interacts with two pharmacologically disctinct binding sites. Indeed, OmIA and its analogues displayed clearly distinct high and low affinity binding sites, with the low affinity binding state more rapidly occupied by OmIA and its analogues, while the high affinity binding site required longer incubation times to develop. This biphasic behavior of [V10Q]OmIA and [V10L]OmIA was not as apparent for other OmIA analogues, possibly due to their greater preference for high-affinity binding site over the low-affinity binding site.

The complex pharmacological profile of OmIA and analogues seen at PAM-α7 nAChRs, with different PAMs differentially enhancing this phenomenon at α7 nAChRs, suggests a direct involvement of type II PAMs in modulating this phenomenon. In contrast, at α3β4 nAChRs, any surmountable antagonism of [V10Q]OmIA and [V10L]OmIA or the biphasic behavior of OmIA are less apparent. The effect of PAMs on OmIA pharmacology may arise from enhanced channel gating and associated conformational changes in the orthosteric ligand binding site induced by PNU120596 and TQS (Barron et al., 2009) (Supplementary Figure S4). Long side chains at position 10 appear to change the way OmIA interacts with PAM-modified orthosteric binding site of α7 nAChRs, although in the absence of PAMs, [V10Q]OmIA docked similarly to OmIA at the α7 nAChR (Supplementary Figure S5). Interestingly, substitution of long side chain at position 10 enhanced PnIA potency and shift it to an agonist at the [L247T]α7 nAChR, a mutant with prolonged desensitization reminiscent of the effect of type II PAMs on nAChR function (Hogg et al., 2003). Further studies are required to determine the extent different side chains at position 10 can stabilise different functional states of the receptor.

In summary, we report the crystal structure of OmIA with *Ls*-AChBP and a model of α-conotoxin OmIA complexed with α7 nAChRs that explains its high potency at α7 nAChRs. OmIA displayed functional insurmountable antagonism at human α7 nAChRs despite a binding to the othosteric site. OmIA pharmacology provides significant new insights into mechanisms of inhibition of α7 nAChRs, and the influence of type II PAMs on nAChR function. These results may facilitate the design of α-conotoxin analogues with novel features and potentially innovative therapeutic leads.

## Data Availability

The datasets presented in this study can be found in online repositories. The names of the repository/repositories and accession number(s) can be found below: wwPDB Deposition; PDB ID 7N43.

## Abbreviations

nAChRs, nicotinic acetylcholine receptors; AChBP, acetylcholine binding protein; *Ls*, *Lymnaea Stagnalis*; Trt, trityl; Acm, S-acetamidomethyl; ACN, acetonitrile; MeOH, methanol; TFA, trifluoroacetic acid; Ub, Ubiquitin; IMAC, immobilized metal affinity chromatography purification; CD, circular diochroism; PNU120596, N-(5-Chloro-2,4-dimethoxyphenyl)-N′-(5-methyl-3-isoxazolyl)-urea; CI, confidence interval; PAMs, positive allosteric modulators; I_2_, iodine; RP-HPLC, reverse phase- high performance liquid chromatography.
