# Supplementary material for: Unique Pharmacological Properties of α-Conotoxin OmIA at α7 nAChRs
Source: Front Pharmacol. 2021 Dec 8;12:803397. doi: 10.3389/fphar.2021.803397 (PMC8692984; doi:10.3389/fphar.2021.803397)
Supplement: Supplementary file 1 [file DataSheet1.pdf]

# Unique pharmacological properties by α-conotoxin OmIA at α7 nAChRs

**Thao N.T. Ho, Nikita Abraham, Richard J. Lewis***

Centre for Pain Research, Institute for Molecular Bioscience, The University of Queensland, St Lucia, QLD, Australia

*** Correspondence:**
Richard J. Lewis
r.lewis@uq.edu.au

**Running title:** Unique pharmacological properties by α-conotoxin OmIA

**Keywords: α-conotoxin, α7 nAChRs, pharmacology, AChBP.**

## SUPPLEMENTARY INFORMATION

**Supplementary Table S1: Data collection and refinement statistic**

| | ***Ls*-AChBP- OmIA** |
|---|---|
| **Data collection** | |
| Space group | $P4_212$ |
| Cell dimensions, Å | a= 169.36 Å, b= 169.36 Å, c= 124.34 Å |
| Cell dimensions, ° | α=90°, β=90°, γ=90° |
| Resolution, Å | 49.19-2.47 (2.53-2.47) |
| Rsym | 0.115 (1.153) |
| I/Iσ | 17.9 (2.0) |
| Completeness (%) | 99.6 (94.2) |
| Multiplicity | 26.9 (25.6) |
| Total no. of reflections | 1748096 (109721) |
| Unique reflections | 65087 (4285) |
| **Refinement** | |
| Resolution Å | 49.19-2.47 |
| Rwork/Rfree | 0.201/ 0.247 |
| rmsd bond distance, Å | 0.009 |
| rmsd bond distance, Å | 1.2 |
| Average B-factor | 70.0 |

**Supplementary Table S2: Receptor-ligand interactions observed in the OmIA/*Ls*-AChBP co-crystal structure**

| OmIA | Principal (+) | Distance ± SEM (Å) | Complementary (-) | Distance ± SEM (Å) |
|---|---|---|---|---|
| Gly1 | Tyr185 | 4.22 ± 0.19 | Glu163 | 2.64 ± 0.16 |
| Cys2 | Tyr185<br>Cys187-Cys188 | 4.42 ± 0.17<br>3.90 ± 0.13 | | |
| Cys3 | | | Tyr164 | 3.46 ± 0.27 |
| Ser4 | | | Trp53<br>Glu163 | 3.76 ± 0.22<br>3.56 ± 0.31 |
| His5 | Tyr89<br>Ser142<br>Tyr185<br>Tyr192<br>Glu193<br>Asp194 | 3.32 ± 0.09<br>4.30 ± 0.17<br>3.90 ± 0.23<br>4.42 ± 0.18<br>3.82 ± 0.21<br>4.62 ± 0.15 | | |
| Pro6 | Tyr89<br>Trp143 | 4.22 ± 0.13<br>3.64 ± 0.13 | Trp53<br>Met114 | 3.86 ± 0.11<br>4.40 ± 0.20 |
| Ala7 | Ser142<br>Trp143<br>Thr144<br>His145<br>Tyr192 | 4.78 ± 0.26<br>4.78 ± 0.15<br>4.20 ± 0.37<br>4.70 ± 0.23<br>3.88 ± 0.04 | | |
| Cys8 | Tyr192 | 3.66 ± 0.07 | | |
| Asn9 | | | Lys34<br>Trp53<br>Gln55<br>Met114<br>Tyr164 | 5.0 ± 0.23<br>>5.0<br>3.58 ± 0.07<br>4.00 ± 0.13<br>3.72 ± 0.26 |
| Val10 | Trp143<br>Thr144 | 3.70 ± 0.30<br>4.52 ± 0.10 | Arg104<br>Leu112<br>Met114 | 4.50 ± 0.08<br>4.92 ± 0.07<br>4.92 ± 0.07 |
| Asn11 | Thr144<br>Glu149<br>Tyr192 | 3.64 ± 0.36<br>>5.0<br>4.04 ± 0.22 | Gln73<br>Arg104 | 3.46 ± 0.30<br>3.68 ± 0.16 |
| Asn12 | Cys188<br>Tyr192 | 3.76 ± 0.12<br>3.56 ± 0.15 | | |
| Pro13 | | | Val106<br>Glu110<br>Leu112 | 4.5 ± 0.10<br>3.74 ± 0.15<br>4.14 ± 0.07 |
| His14 | | | | |
| Ile15 | Cys187 | 4.14 ± 0.23 | | |
| Cys16 | | | Gln55 | 4.22 ± 0.29 |
| Gly17 | | | Gln55<br>Thr155 | 4.24 ± 0.34<br>4.18 ± 0.35 |

**Supplementary Table S3. Amino acid sequences of OmIA and variants**

| Peptide name | Peptide sequence | Theoretical mass | Observed mass |
|---|---|---|---|
| OmIA | G**C**CSHPA**C**NVNNPHI**C**G* | 1719.45 | 1719.24 |
| [H5R]OmIA | G**C**CSRPA**C**NVNNPHI**C**G* | 1740.23 | 1740.26 |
| [N9H]OmIA | G**C**CSHPA**C**HVNNPHI**C**G* | 1744.16 | 1744.34 |
| [V10Q]OmIA | G**C**CSHPA**C**NQNNPHI**C**G | 1749.24 | 1749.95 |
| [N11D]OmIA | G**C**CSHPA**C**NVDNPHI**C**G* | 1721.49 | 1721.50 |
| [V10T]OmIA | G**C**CSHPA**C**NTNNPHI**C**G* | 1722.87 | 1722.95 |
| [V10A]OmIA | G**C**CSHPA**C**NANNPHI**C**G* | 1692.64 | 1692.34 |
| [V10L]OmIA | G**C**CSHPA**C**NLNNPHI**C**G* | 1735.13 | 1735.83 |
| [V10K]OmIA | G**C**CSHPA**C**NKNNPHI**C**G* | 1750.24 | 1750.86 |
| [V10E]OmIA | G**C**CSHPA**C**NENNPHI**C**G* | 1750.59 | 1750.23 |

**Supplementary Table S4.** IC$_{50}$ values for displacement of [$^{3}$H]-epibatidine binding on *Ls*-AChBPs and inhibition of choline activation of α7 nAChRs with the addition of nAChR type II PAMs PNU120596, TQS and inhibition of nicotine activate of α3β4 nAChRs in SH-SY5Y cells by OmIA and its analogues at position 10. *Ratios were calculated between OmIA and its analogues. Data represent mean ± SEM of triplicate data from three independent experiments. [a]denotes significant difference in IC$_{50}$ values to wildtype OmIA ($p < 0.05$). [b]denotes 95% CI of curve bottom values non-overlapping 0%.

| | [$^{3}$H]-epibatidine binding IC$_{50}$ ± SEM (μM) | | FLIPR SH-SY5Y IC$_{50}$ ± SEM (μM) | | | | | |
|---|---|---|---|---|---|---|---|---|
| | *Ls*-AChBP | Ratio* | PNU120596-α7 nAChRs | Ratio* | TQS-α7 nAChRs | Ratio* | α3β4 nAChRs | Ratio* |
| OmIA | 0.28 ± 0.07 | 1 | 0.27 ± 0.02 | 1[b] | 0.50 ± 0.09 | 1[b] | 0.16 ± 0.03 | 1[b] |
| [V10A]OmIA | 1.0 ± 0.58 | 3.57[a] | 0.29 ± 0.02 | 1[b] | 0.68 ± 0.07 | 1[b] | 0.15 ± 0.01 | 1[b] |
| [V10T]OmIA | 1.0 ± 0.05 | 3.57[a] | 0.71 ± 0.11 | 2.63[a,b] | 3.40 ± 0.99 | 6.80[a,b] | 0.88 ± 0.23 | 5.50[a,b] |
| [V10L]OmIA | 0.68 ± 0.13 | 2.42 | 0.28 ± 0.01 | 1[a] | 0.05 ± 0.01 | 0.01[a] | 0.29 ± 0.04 | 1.81 |
| [V10E]OmIA | >10 | >10[a] | >10 | >10[a] | >10 | >10[a] | 0.92 ± 0.16 | 5.75[a] |
| [V10K]OmIA | >10 | >10[a] | >10 | >10[a] | >10 | >10[a] | 0.88 ± 0.23 | 5.50[a] |

**Supplementary Table S5**. Effect of OmIA and its analogues at position 10 on choline concentration-activation curve at α7 nAChRs with the addition of nAChR type II PAMs, PNU120596 and TQS, and nicotine concentration-activation curve at α3β4 nAChRs in SH-SY5Y cells. Data represent mean ± SEM of triplicate data from three independent experiments. [b]denotes 95% CI of curve top values non-overlapping 100%. [c] denotes significant difference in $EC_{50}$ values to agonist alone ($p < 0.05$)

| Peptide concentration | | Choline at PNU120596-α7 nAChRs (μM) | Choline at TQS-α7 nAChRs (μM) | Nicotine at α3β4 nAChRs (μM) |
|---|---|---|---|---|
| Choline | | 9 ± 0.89 | 3 ± 0.26 | |
| Nicotine | | | | 8 ± 0.35 |
| [V10T]OmIA | 0.3μM | | | 10 ± 0.60[b] |
| | 1μM | 8 ± 1.40[b] | 3 ± 0.63[b] | 9 ± 1.50[b] |
| | 3μM | 12 ± 0.28[b] | 3 ± 0.11[b] | 10 ± 0.67[b] |
| | 10μM | 11 ± 1.80[b] | 3 ± 0.59[b] | |
| [V10A]OmIA | 0.1μM | | | 10 ± 0.85[b] |
| | 0.3μM | 7 ± 1.20[b] | | 11 ± 1.10[b] |
| | 1μM | 8 ± 0.47[b] | 3 ± 0.20[b] | 12 ± 0.56[b] |
| | 3μM | 11 ± 1.20[b] | 4 ± 1.00[b] | |
| | 10μM | | 5 ± 0.48[b] | |
| [V10L]OmIA | 0.1μM | | 6 ± 1.50[b] | 9 ± 0.19[b] |
| | 0.3μM | 9 ± 0.67[b] | 13 ± 2.50[b] | 9 ± 0.81[b] |
| | 1μM | 18 ± 1.20[b,c] | 28 ± 6.00[b] | 13 ± 4.40[b] |
| | 3μM | 38 ± 13.30[b,c] | 36 ± 5.00[b] | |

```
Ls-AChBP --LDR---ADILYNIRQTSRPDVIPTQRDRPVAVSVSLKFINILEVNEITNEVDVVFWQQ 55
hα7      GEFQRKLYKELVKNYNPLERP---VANDSQPLTVYFSLSLLQIMDVDEKNQVLTTNIWLQ 57

Ls-AChBP TTWSDRTLAWNSSHSPDQ--VSVPISSLWVPDLAAYNAISKPEVLTPQ-LARVVSDGEVL 112
hα7      MSWTDHYLQWNVSEYPGVKTVRFPDGQIWKPDILLYNSADERFDATFHTNVLVNSSGHCQ 117

Ls-AChBP YMPSIRQRFSCDVSGV-DTESGATCRIKIGSWTHHSREISVDPTTENSDDSEYFSQYSRF 171
hα7      YLPPGIFKSSCYIDVRWFPFDVQHCKLKFGSWSYGGWSLDLQM--QEADISG-YIPNGEW 174

Ls-AChBP EILDVTQKKNSVTYSCCPEAYEDVEVSLNFRKKGRSEIL 210
hα7      DLVGIPGKRSERFYECCKEPYPDVTFTVTMRRRT----- 208
```

**Figure S1. Sequence alignment of *Ls*-AChBP and human α7 nAChRs.** The human (Uniprot: P36544) is aligned with the *Ls*-AChBP

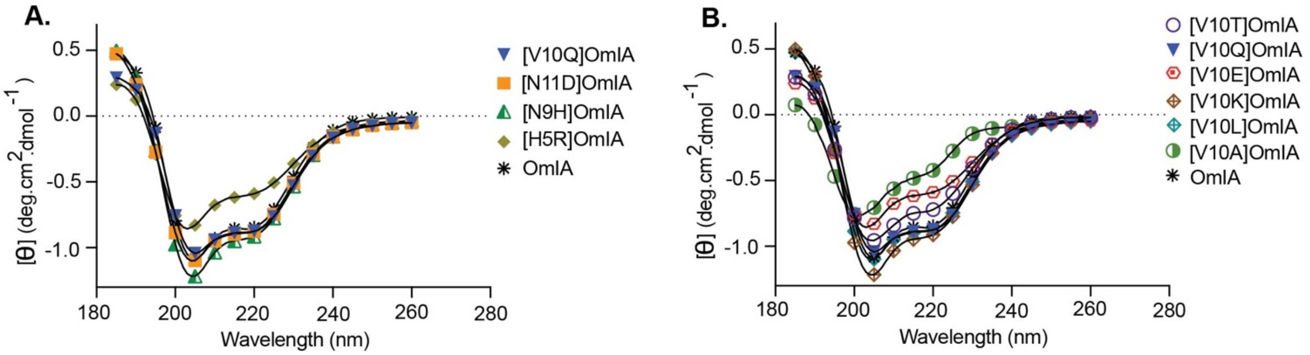


**Figure S2. CD spectra of OmIA and its analogues (A) and analogues at position 10 (B)**

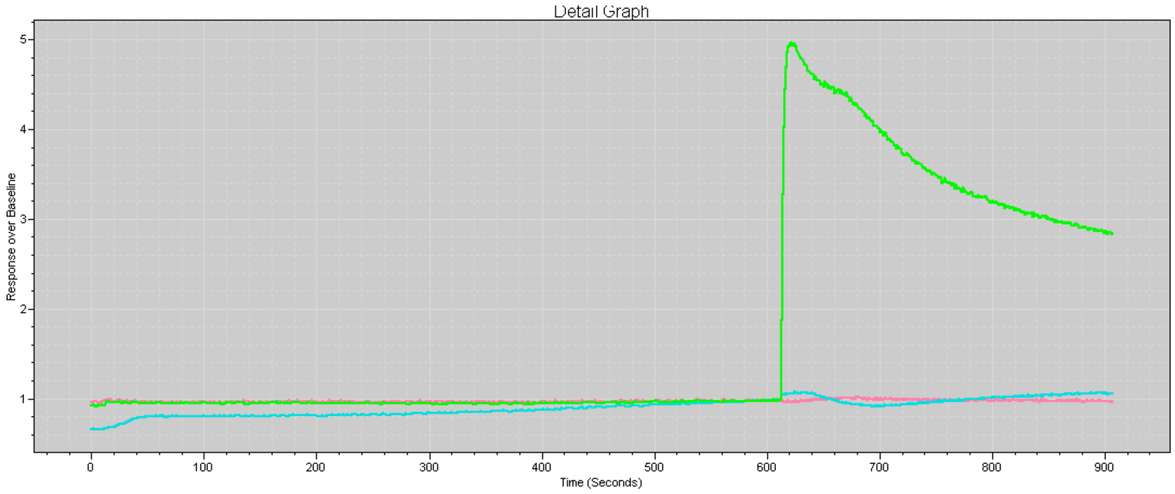


**Figure S3**. FLIPR traces showing OmIA (10uM) inhibition of choline responses at human α7 nAChRs. OmIA added at time 600s did not elicit a $Ca^{2+}$signal at human α7 nAChRs in the presence of PNU120596 but fully inhibited choline responses (cyan trace). Choline added at 600s induced a clear agonist response at human α7 nAChRs (green trace) over the buffer control (pink trace).

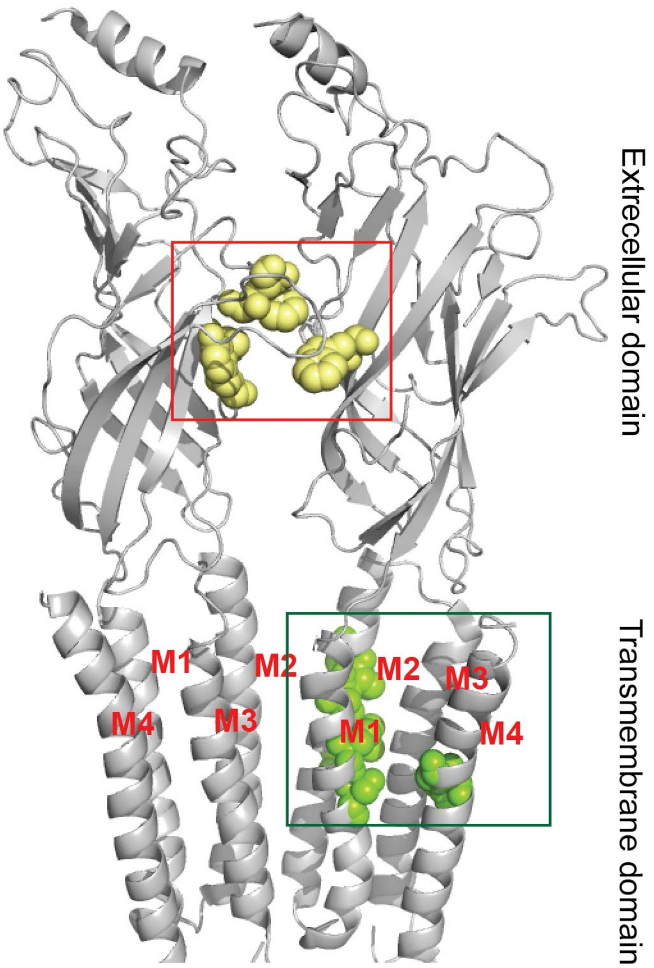


**Figure S4. Structural domains of nAChR regulating PAM activity and/or binding using *Torpedo* acetylcholine (ACh) (2BG9).** This view shows two subunits each with four transmembrane helixes (M1–M4) arranged clockwise with the M2 helix facing the channel pore. The orthosteric binding site for agonists in extracellular domain is displayed as yellow spheres under loop C (red box). Residues involved in the binding of α7 type II PAMs, PNU-120596 and TQS, potentiation are displayed as green spheres (green box).

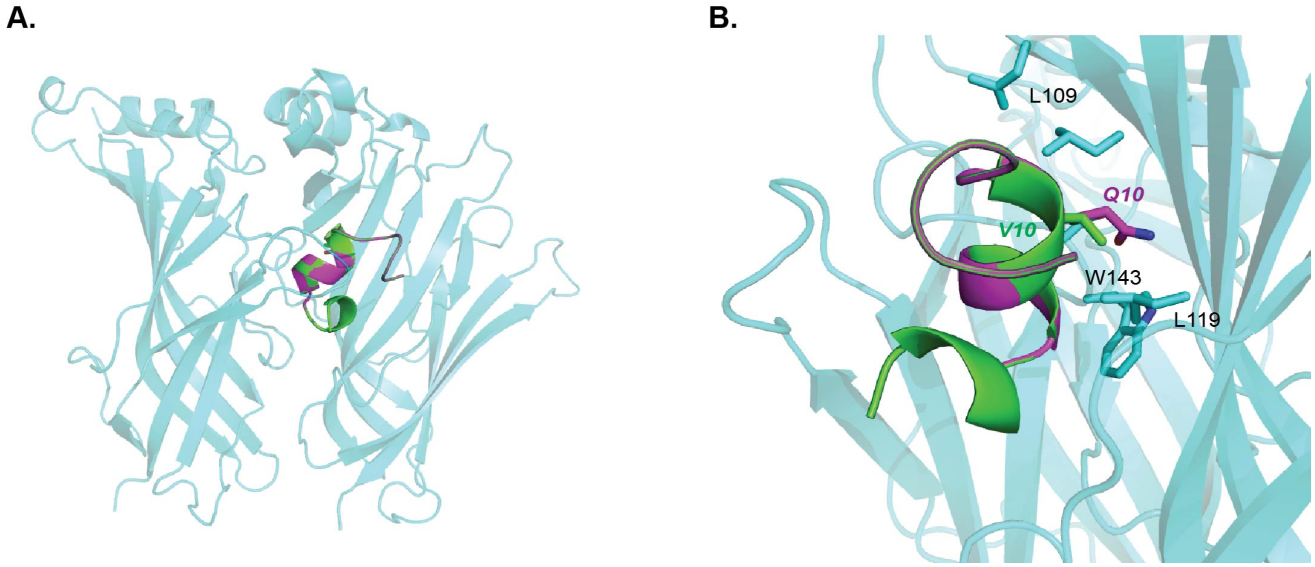


**Figure S5. The docking model of [V10Q] human α7 nAChRs.** (A) [V10Q]OmIA (magneta) docked similarly to OmIA (green) at the α7 nAChR. (B) OmIA_Val10 and [V10Q]OmIA _Gln10 showed similar interactions at the binding pocket with residues on both the principal and complementary face.
